# Detection of Driver Drowsiness Using Wavelet Analysis of Heart Rate Variability and a Support Vector Machine Classifier

**DOI:** 10.3390/s131216494

**Published:** 2013-12-02

**Authors:** Gang Li, Wan-Young Chung

**Affiliations:** Department of Electronic Engineering, Pukyong National University, Busan 608-737, Korea; E-Mail: ligang@pknu.ac.kr

**Keywords:** driver fatigue, heart rate variability, wavelet decomposition, support vector machine, photoplethysmography, Bluetooth, smartphone, internet

## Abstract

Driving while fatigued is just as dangerous as drunk driving and may result in car accidents. Heart rate variability (HRV) analysis has been studied recently for the detection of driver drowsiness. However, the detection reliability has been lower than anticipated, because the HRV signals of drivers were always regarded as stationary signals. The wavelet transform method is a method for analyzing non-stationary signals. The aim of this study is to classify alert and drowsy driving events using the wavelet transform of HRV signals over short time periods and to compare the classification performance of this method with the conventional method that uses fast Fourier transform (FFT)-based features. Based on the standard shortest duration for FFT-based short-term HRV evaluation, the wavelet decomposition is performed on 2-min HRV samples, as well as 1-min and 3-min samples for reference purposes. A receiver operation curve (ROC) analysis and a support vector machine (SVM) classifier are used for feature selection and classification, respectively. The ROC analysis results show that the wavelet-based method performs better than the FFT-based method regardless of the duration of the HRV sample that is used. Finally, based on the real-time requirements for driver drowsiness detection, the SVM classifier is trained using eighty FFT and wavelet-based features that are extracted from 1-min HRV signals from four subjects. The averaged leave-one-out (LOO) classification performance using wavelet-based feature is 95% accuracy, 95% sensitivity, and 95% specificity. This is better than the FFT-based results that have 68.8% accuracy, 62.5% sensitivity, and 75% specificity. In addition, the proposed hardware platform is inexpensive and easy-to-use.

## 1. Introduction

Driving while fatigued is just as dangerous as drunk driving and may result in car accidents. It is easy to detect drunkenness using alcohol sensing devices, but no reliable, inexpensive, and easy-to-use device for detecting driver drowsiness exists. The standard clinical tests for measuring drowsiness are the Multiple Sleep Latency Test (MSLT) and the Maintenance of Wakefulness Test (WMT) combined with polysomnography (PSG) datasets [1]. These measurements are very expensive and cumbersome to perform. It is also impossible to apply these methods to the task of detecting driver drowsiness in actual driving environments. For instance, wearing multiple sensors is uncomfortable for the driver and may also impede the driver's movements.

Photoplethysmography (PPG) is a low-cost and noninvasive means of sensing the cardiovascular blood volume pulse through variations in transmitted or reflected light [2]. Therefore, if driver drowsiness can be detected using only PPG recordings, it will be possible to detect driver drowsiness simply, inexpensively, and less intrusively. Several previous studies have concluded that the heart rate (HR) varies significantly between the alert state and drowsy state [3,4]. Furthermore, several studies have confirmed that HRV-based methods are able to recognize driver drowsiness [5–9]. HRV signals are defined as the constant change of the interval between heart rate. In general, HRV signals are easily obtained and can be used as indicators of the responses of the autonomic nervous system (ANS) to stress, drowsiness, and other related factors, because the ANS is influenced by the sympathetic nervous system (SNS) and the parasympathetic nervous system (PNS). HRV signals are usually calculated by analyzing a time series of beat-to-beat intervals that are measured by an electrocardiography (ECG) or derived from a pulse wave signal that is measured using the PPG waveform. In the frequency domain, the HRV is usually grouped into very low frequency (VLF: 0.003–0.04 Hz), low frequency (LF: 0.04–0.15 Hz), and high frequency (HF: 0.15–0.4 Hz) by means of FFT-based power spectrum density (PSD). It is worthwhile to note that FFT is only applied to equidistantly sampled series so that the raw HRV time series needs to be converted to equidistantly sampled series by interpolation methods prior to FFT analysis [10,11]. In this study, the cubic spline interpolation was used. The LF/HF ratio is defined as the ratio of power in LF band to power in HF band [10]. There is a strong relationship between the LF/HF ratio and the driver's fatigue level [8], although the findings related to LF/HF ratio are less consistent. For example, Shin *et al.* [5], Mahachandra *et al.* [6], and Jiao *et al.* [7] all concluded that the LF/HF ratio increases when driver drowsiness occurs, while Yang *et al.* [8] and Patel *et al.* [9] found that the LF/HF ratio decreases progressively as the driver progresses from an alert state to a drowsy state.

Despite the impressiveness of LF/HF ratio monitoring, a common drawback of the previous studies is that HRV signals have been regarded as stationary signals with frequencies that did not vary over time. Additionally, the FFT method, which is the most well-known method for analyzing stationary signals, was usually used to generate the features (e.g., LF/HF ratio) that are used for further classification. However, in actual working environments, drivers often try to remain alert even though they are feeling sleepy already. Thus, the HRV dynamics for drowsy drivers are complex, non-stationary, and changing over time. It is not uncommon to use non-stationary analysis methods, such as the wavelet analysis, when studying physiological signals. For example, Jahankhani *et al.* [12] used wavelet-based electroencephalograph (EEG) features to diagnose epilepsy. Khandoker *et al.* [13] used wavelet multi-scale analysis to estimate the risk of falls for the elderly. In addition, in 2009, Khandoker *et al.* [14] conducted a study about the automated detection of obstructive sleep apnea using ECG signal and wavelet transform. Haddad *et al.* [15] concluded that most physiological signals are non-stationary signals. In the field of driver drowsiness detection, several studies have successfully extracted wavelet-based features from EEG signal [16], eyelid signals [17] and even steering wheel movements [18]. In a more recent work, a hybrid algorithm using EEG, electrooculogram (EOG), ECG and wavelet-packet-based feature is addressed for driver drowsiness detection [19]. The combination of three physiological signals achieved an overall classification accuracy of 97% for all subjects. However, there has been little research that has focused on the non-stationary analysis of HRV signals, especially in the field of driver fatigue detection. Regarding the design of the classifier (a mathematical model used to classify drowsy and alert events), the Bayesian network (BN) was used in our previous studies [20,21]. The BN is based on the posterior probabilities of training data. Thus, it can provide early detection results as compared to general linear or nonlinear classifiers. However, the calculation of posterior probabilities depends on a conditional probability table that is based on time-consuming, empirical studies. Recently, SVM has emerged as a powerful technique for pattern recognition. The primary advantage of SVM is its ability to minimize both structural and empirical risk [22], thereby leading to better generalizations for new data classifications, even with limited training datasets. Thus, in this study, our goal is to assess whether a method that uses wavelet-based features of HRV can detect driver drowsiness more effectively than methods that use the conventional FFT-based LF/HF ratio. The aim is to develop a reliable driver drowsiness detection system that combines SVM with an inexpensive and easy-to-use hardware platform. We will also include a built-in alertness boosting application in our solution.

## 2. Proposed Hardware Platform

**Figure 1.**
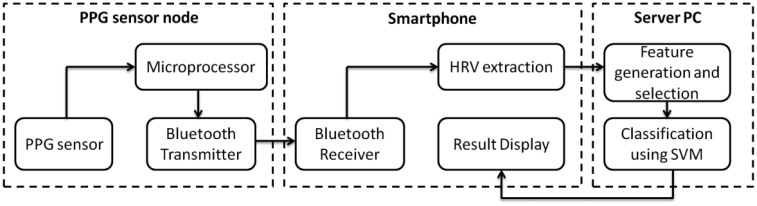
Schematic diagram of the proposed driver drowsiness detection system.

Figure 1 shows the system configuration, which is comprised of a PPG sensor, a microprocessor unit (MCU), a wireless transmitter, a smartphone, and a server PC that connects to the internet. The wireless PPG sensor incorporates an MCU and a Bluetooth module and can be attached to the steering wheel. The outputs from the PPG sensor node are transmitted wirelessly via Bluetooth communications to a smartphone that extracts the HRV time series. After this, the smartphone [Transmission Control Protocol (TCP) client] transmits the HRV signals to an external PC (TCP server) for feature generation, feature selection, and classification. Finally, the classification result is fed back to a friendly user interface (smartphone) for self-monitoring or activating the alarm and the built-in alertness boosting solution.

### 2.1. PPG Sensor Node

There are two types of probes used in medical instruments for PPG measurements. The first type is a transmission probe that has an emitter on the opposite side from the detector. The second type is known as a reflection probe. This type of probe has an emitter that is on the same side as the detector. An infrared (IR) light is transmitted from the emitter and the IR signal is received by the photo detector through the skin and veins. Reflection type PPG sensors were chosen for this study because they are more convenient for drivers. The PPG sensor can be mounted on the steering wheel and can capture the PPG readings directly from a finger that is resting on the steering wheel. The reflection type PPG sensors are less intrusive and, unlike transmission type sensors, do not cause discomfort for the driver. We chose the Laxtha RP520 PPG sensor (Laxtha, Daejeon, Korea). Based on the requirements of low-cost and low-power consumption, the open-source LilyPad Arduino hardware platform (SparkFun Electronics, Boulder, CO, USA) was selected. This open hardware platform is designed for wearables and e-textiles [23]. Thus, it can be easily attached to the steering wheel. Table 1 shows the specifications of the proposed PPG sensor node.

### 2.2. Smartphone

Smartphones have high-speed data transmission capabilities (e.g., 3G, 4G) and have embedded microprocessors with capabilities, such as Bluetooth and WiFi, for connecting wirelessly to external devices. For this study, the Samsung Galaxy SIII (Android 4.1.2) smartphone was used as a reliable and user-friendly Bluetooth-to-Internet gateway. It was also used to display the raw PPG signals and to extract 1-min HRV time series. Table 2 displays the specific steps in the HRV extraction procedure, where a 1st-order differential operation is used to remove the artifacts when drivers rotate the steering wheel and re-sampling is used to up-sample the raw HRV time series, in order to generate enough samples for FFT analysis. The 1st-order differential operation *v*(*x*) of PPG signal *y*(*x*) in discrete time can be demonstrated by Equation (1):

(1) $$v(x)=\frac{1}{\Delta t}(y(x)-y(x-1))$$

where *x* is the total number of samples and Δ*t* is the sampling rate.

After the HRV extraction, the smartphone sends the HRV signals to an external computer via an Internet connection for secondary signal processing, including the calculation of LF/HF ratios, the generation and selection of wavelet-based features, and the classification process via the SVM. Next, the classification result can be sent back to the smartphone to enable the driver to self-monitor. If the driving condition is classified as “fatigued”, a “Searchnearby” service, which is based on the Google Map Application Interface (API) and the Google Place API, is activated so that the driver can stop at the nearest coffee shop and drink a coffee and boost alertness. Drinking coffee is not uncommon among drivers. For example, the British Broadcasting Corporation (BBC) reported this year that long-distance lorry drivers who drink coffee have fewer road traffic accidents [27].

### 2.3. Server PC

The server PC uses vb.NET application software (Microsoft Corporation, Redmond, WA, USA), MATLAB^®^ application software (Mathworks, Natick, MA, USA), and IBM SPSS Statistics software (IBM, Armonk, NY, USA) (a commercial statistical analysis tool). The main purpose of using vb.NET is to receive HRV time series from the Internet (smartphone). The MATLAB^®^ application is responsible for feature generation using the FFT and wavelet decomposition methods and for feature classification using the SVM. The combination of vb.NET and MATLAB^®^ application can be easily realized using Matlab Builder™ NE. As a result, the vb.NET application is able to make direct use of the math and data analysis functions that are built into MATLAB^®^. The SPSS is used to perform ROC analysis for feature selection.

## 3. Proposed Algorithm

**Figure 2.**
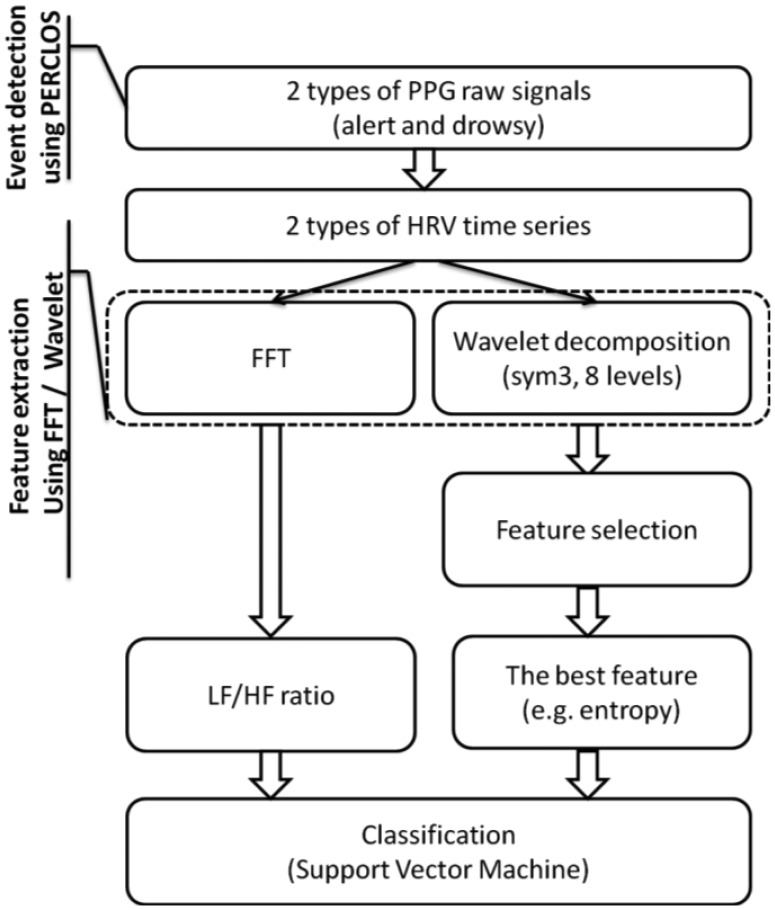
Flowchart of the proposed method for classifying the driver as alert or drowsy based on the PPG signal and the comparison of the FFT and wavelet analyses.

Figure 2 shows the schematic diagram of the proposed algorithm, where the input is a PPG signal and the output is the classification of the driver as alert or drowsy.

### 3.1. Event Detection Using PERCLOS

First, the PPG input signal is divided into 1-min intervals and the two driving events are verified based on the average percentage of eyelid closure over pupil over time (PERCLOS) measurements over the interval. Detailed information about the calculation of PERCLOS can be found in our earlier studies [20,28]. Table 3 describes the specific characteristics of the alert and drowsy driving conditions, where a PERCLOS value of 0%∼30% indicates alert conditions and 30%∼40% indicates drowsy conditions. This classification criterion was set through our pilot study when subjects reported their sleepiness states using the Karolinska sleepiness scale (KSS) [29]. For example, subjects rated their KSS results as #9 (sleepy, some effort to keep alert) when PERCLOS was 30%∼40%. The KSS measures the subjective level of sleepiness at a particular time during the day. On this scale subjects indicate which level best reflects the psycho-physical state experienced in the last 10 min [29]. This is why we collected data for 10 min (for more details please refer to Table 5 in the Results section).

### 3.2. Feature Extraction Using FFT and Wavelet Decomposition

Next, the FFT-based and wavelet-based feature extractions are performed. A discrete wavelet transform (DWT), which is based on the Symlet mother wavelet with order 3, is used to extract the features of the HRV time series. The DWT gives a decomposition of a given signal into a set of approximate (A*_i_*) and detailed (D*_i_*) coefficients of level *i* (*i* = 1, …, *n*). The frequency range of each level is calculated as shown in Equation (2), where *n* is the index of level and *f_s_* is the re-sampling rate for the HRV time series:

(2) $$\text{Frequency}\_\text{range}=(\frac{1}{2^{n+1}}\sim \frac{1}{2^{n}})\times f_{s}$$

In order to compare with classical HRV frequency analysis, each HRV signal is decomposed into eight levels, the frequency range of which is shown in Table 4. For each level the Shannon's entropy, mean, variance, kurtosis, and spectral component β are extracted from D*_i_* (*i* = 1, …, 8) and A_8_[13,14,22]. In total, it is possible to obtain 43 wavelet-based features from each 1-min HRV time series. Since the standard shortest duration for LF/HF analysis on HRV is 2 min [10], the LF/HF ratio is calculated for 2-min durations. For reference purposes, the 1-min and 3-min HRV signals are also used to calculate LF/HF ratio, as well as selected wavelet-based features.

### 3.3. Feature Selection Using ROC Analysis

In order to obtain the relative importance of features, ROC analysis was used [30]. The area under the ROC curve is called ROC_area_ and can be used as an effective criterion for design a classifier [13,14,22]. Using ROC analysis, the LF/HF ratio and the best wavelet-based feature with higher ROC_area_ are selected to form feature vectors for training the SVM, respectively. In SPSS, the ROC analysis requires at least two feature vectors. One feature vector is called the “state variable” which indicates the verified classification labels. The other feature vector is called the “test variable” and contains the wavelet-based feature vectors or FFT-based feature vectors. For two-class feature selection, the “state variable” contains two values (e.g., 1: drowsy and −1: alert). The ROC_area_ value can be any value from 0 to 1. If the mean of the feature values from drowsy group is higher than the alert group, then a ROC_area_ value of 1.00 means that the features are exactly separable. If the mean of the drowsy group is lower than alert group, then a ROC_area_ value of 0.00 means that the features are exactly separable. A ROC_area_ value of 0.50 implies that the features are completely overlapped and thus non-separable. In this case, a ROC_area_ value of 0.7 (or 0.3) implies that the features are acceptable for classification [14].

### 3.4. Classification Using SVM

**Figure 3.**
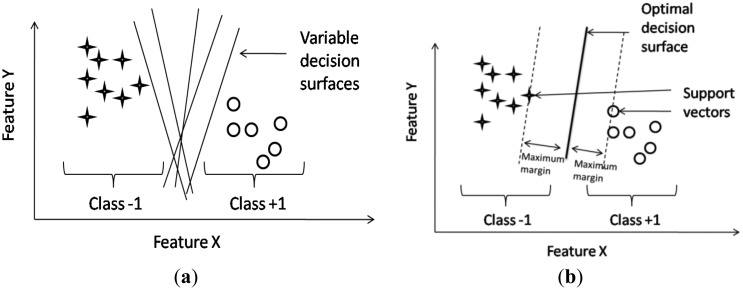
Example of two-class (+1 & −1) problem. The circles and stars represent samples of class +1 and −1, respectively. (**a**) multiple decision surfaces and (**b**) optimal decision surface.

In this study, the SVM is used to automatically recognize drowsy driving events. SVM theory has a long history of development starting from the early 1950s [31]. SVM, introduced by Vapnik and Cortes in 1995 [31], is more powerful and already packaged in some analysis tools, such as MATLAB^®^. Just like any other classifiers, the aim of SVM is to find a decision surface that splits the dataset into two parts. All data lying on one side of the decision surface will be classified as members of one class and all data lying on the other side of the decision surface will be classified as members of another class. However, this kind of decision surface is not unique (see Figure 3a). It follows the difference between SVM and other classifiers: SVM is able to find the unique decision surface which also has a maximum distance or margin between the two datasets. That is to say, SVM is able to find the optimal decision surface. Figure 3b is an example with two-dimensional data where each data is represented by two features. Actually, SVM theory is particularly helpful for higher-dimensional feature space, which cannot be made such intuitive drawings.

In brief, the theory of SVM introduced by Vapnik and Cortes is as follows [31]: assume that the input dataset is represented by N n-dimensional data points *x⃗*_1_,*x⃗*_2_,…,*x⃗_N_* ∈ **ℜ***^n^* and corresponding labels *y*_1_, *y*_2_, …, *y*_n_ ∈ {−1,+1}, SVM maps each point *x⃗_i_* from the input space **ℜ***^n^* to the feature space **H** by means of the mapping function Φ(*x⃗_i_*) and finds a linear decision surface to classify the negative data points and the positive ones in the feature space. The linear decision surface is defined as Equation (3) with constraint Equation (4):

(3) $$\vec{w}\cdot \Phi (\vec{x})+b=0$$

(4) $$y_{i}(\vec{w}\cdot \Phi ({\vec{x}}_{i})+b)\geq 1\cdot \forall i$$

where the *w⃗* is a vector perpendicular to the decision surface and *b* is a scalar (decision surface bias). In order to maximize the margin of separation between the classes ( $\frac{2}{\left\|\vec{w}\right\|}$ or equivalent to minimize $\frac{1}{2}{\left\|\vec{w}\right\|}^{2}$), SVM constructs a unique decision surface by applying Lagrange multiplier and transforming into the following dual problem:

(5) $$\underset{\lambda }{\min}(\frac{1}{2}\sum_{j,k=1}^{N}\lambda_{j}\lambda_{k}y_{j}y_{k}K({\vec{x}}_{j},{\vec{x}}_{k})-\sum_{j=1}^{N}\lambda_{i})$$

(6) $$\text{subject to}\sum_{i=1}^{N}\lambda_{i}y_{i}=0\;\text{and}\;0\leq \lambda_{i}\leq C\forall i$$

where *λ* = (*λ*_1_, …, *λ_N_*) is the Lagrange multiplier, *C* is a constant parameter which determines the tradeoff between the maximum margin and minimum classification error. In general, *C* has to be selected for the input dataset at hand by the user. *K*(.,.) is denoted as *K*(*x⃗_j_*, *x⃗_k_*) = Φ(*x⃗_j_*) · Φ(*x⃗_k_*), which is so-called kernel function. By using kernel function, SVM does not need to know explicitly the mapping function Φ(*x⃗*): ℜ^*n*^ → **H**; it is sufficient only to know the dot product between mappings of two data points. Having determined the optimum Lagrange multiplier, the optimum solution for the vector *w⃗* is given by:

(7) $$\vec{w}=\sum_{j=1}^{N}\lambda_{j}y_{j}\Phi ({\vec{x}}_{j})$$

Then SVM is able to classify any input *x⃗* using the function:

(8) $$f(\vec{x})=\text{sign}(\vec{w}\cdot \Phi (\vec{x})+b)=\text{sign}(\sum_{j=1}^{N}\lambda_{j}y_{j}K({\vec{x}}_{j},x)+b)$$

In this study, the LF/HF ratio and wavelet-based feature were used as input features to the SVM. The SVM outputs represent the driving types (−1 = alert, +1 = drowsy). Both of linear and non-linear kernel (radial basis function) were studied in order to obtain the highest level of classification accuracy. The parameter *C* and Radial Basis Function parameter γ are optimized using a simple search procedure with γ = {10,1,0.1} and *C* = {10,1,0.1}. In this study, SVM was implemented on the MATLAB^®^ SVM toolbox.

## 4. Results and Discussion

Four subjects participated in this study. The subjects included three males (subjects A, B and C) and one female (subject D). Each of them was tested for 10 min for data collection during an alert state and 10 min for data collection during a drowsy state. A total of 40 alert and 40 drowsy samples were obtained, with each sample having a duration of 1 min. All subjects were tested in a driving stimulation environment which is similar to our previous study [20]. The summary of the subjects' data is given in Table 5.

**Figure 4.**
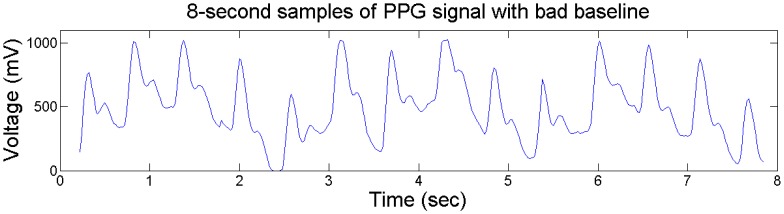
Typical plots of PPG signal with bad baseline.

**Figure 5.**
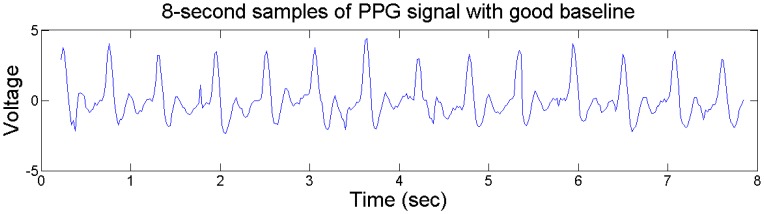
Typical plots of PPG signal with good baseline.

The typical plots of PPG signal before and after the 1st-order differential operation are shown in Figures 4 and 5. We can see that the 1st-order differential operation could effectively remove the artifacts caused by driver's movement on steering wheel, which helps the extraction of peak-to-peak intervals of PPG signals.

**Figure 6.**
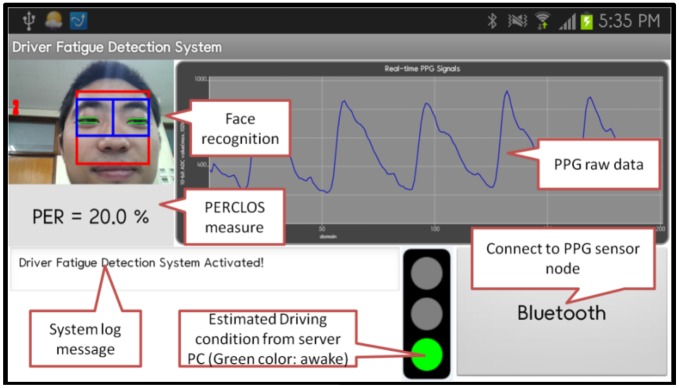
Screenshot of a smartphone that shows the measurement of the PERCLOS, the display of raw PPG data, and the estimation of the driver's level of alertness.

**Figure 7.**
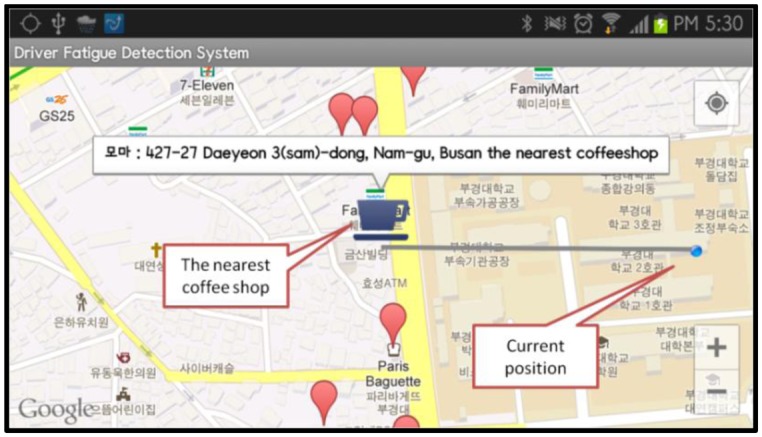
Screenshot of a smartphone that shows a demonstration of the “Searchnearby” service that indicates the location of the nearest coffee shop.

Figure 6 displays the PERCLOS measures and the raw PPG data for subject A when he was alert. Figure 7 shows the alertness boosting solution that is activated when a drowsy driver has been detected.

**Figure 8.**
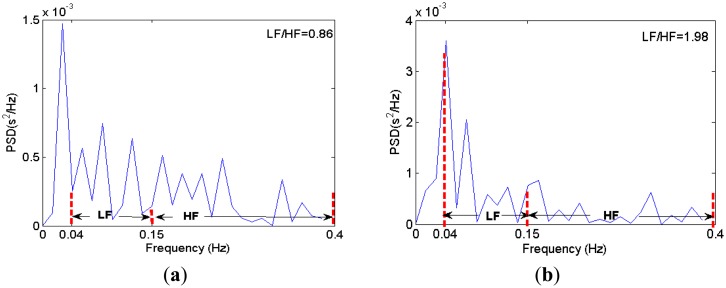
The typical LF and HF power spectrum computed by our server PC. (**a**) represents alert driving; (**b**) represents drowsy driving.

A typical HRV power spectrum for alert and drowsy driving are shown in Figure 8, where we can see that the LF/HF ratio increases when driver drowsiness occurs, which is consistent with the previous results [5–7].

### 4.1. Feature Selection Using ROC_area_

The ROC_area_ for all 43 wavelet-based features from subject A are shown in Table 6. The ROC_area_ values that are higher than 0.7 are in bold and italicized.

**Figure 9.**
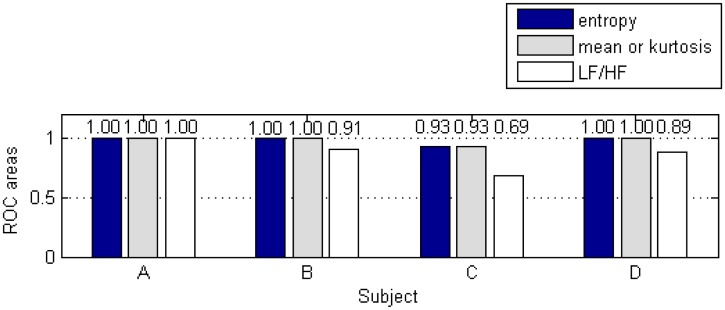
Comparison of LF/HF and wavelet-based features based on standard HRV duration.

Based on the standard shortest duration for FFT-based analysis of HRV signals, the entropy and mean of level A_8_ are the best two wavelet-based features for all of the male subjects. For the female subject, on the other hand, the entropy of level A_8_ and kurtosis of level D_2_ are found to be the best two features. The maximum and minimum ROC_area_ of LH/HF ratio are found for subject A (=1.00) and subject C (=0.69), as shown in Figure 9. The averaged ROC_area_ for the four subjects is 0.87, which is effective for classifying alert and drowsy events. For the wavelet-based features, the entropy and mean (or kurtosis) both have a maximum ROC_area_ (=1.00) for all of the subjects (except for subject C whose ROC_area_ value is slight less than 1.00). The averaged ROC_area_ of entropy for the four subjects is 0.98, which is excellent for classifying alert and drowsy events.

**Figure 10.**
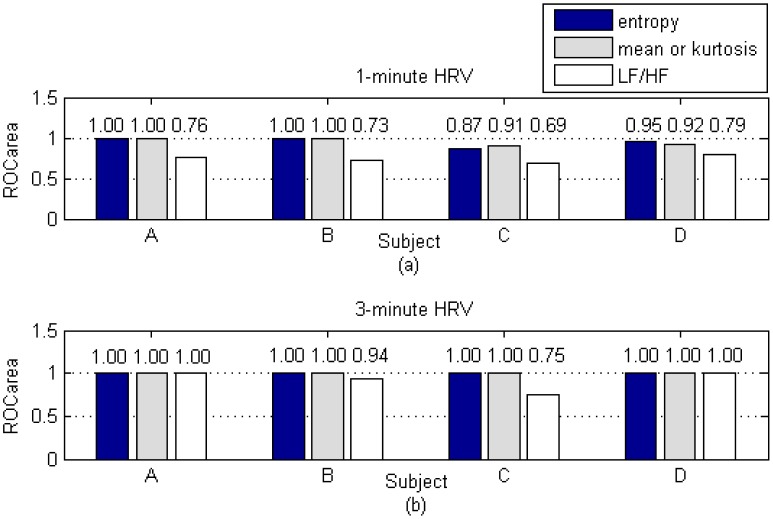
Comparison of LF/HF and wavelet-based features based on 1-min and 3-min HRV durations; (**a**) ROC_area_ for 1-min HRV signals; (**b**) ROC_area_ for 3-min HRV signals.

The ROC_area_ of the LF/HF ratio and the wavelet-based features, which is based on 1-min and 3-min HRV durations, is shown in Figure 10. For both the wavelet-based features and the LF/HF, the ROC_area_ values increase as the HRV durations increase. For example, the entropy and LF/HF for subject C rise from 0.87 and 0.69 for the 1-min HRV duration to outstanding measurements of 1.00 and 0.75 for the 3-min HRV duration. This indicates that the accuracy levels of classifications increase as the HRV durations increase, regardless of what measures are being used, whether wavelet-based features or LF/HF ratios. However, the averaged ROC_area_ for LF/HF is still lower than the average ROC_area_ for wavelet-based features, even when the HRV duration is extended to 3 min. For example, the ROC_area_ of wavelet-based features has reached 1.00 for all subjects, even though half of them still have an LF/HF-based ROC_area_ value that is less than 1.00. More specifically, the averaged ROC_area_ for LF/HF with 3-min HRV signals is still lower than the averaged ROC_area_ for entropy for 1-min HRV signals. This result indicates that the wavelet-based feature gives better performances during real-time classifications.

**Figure 11.**
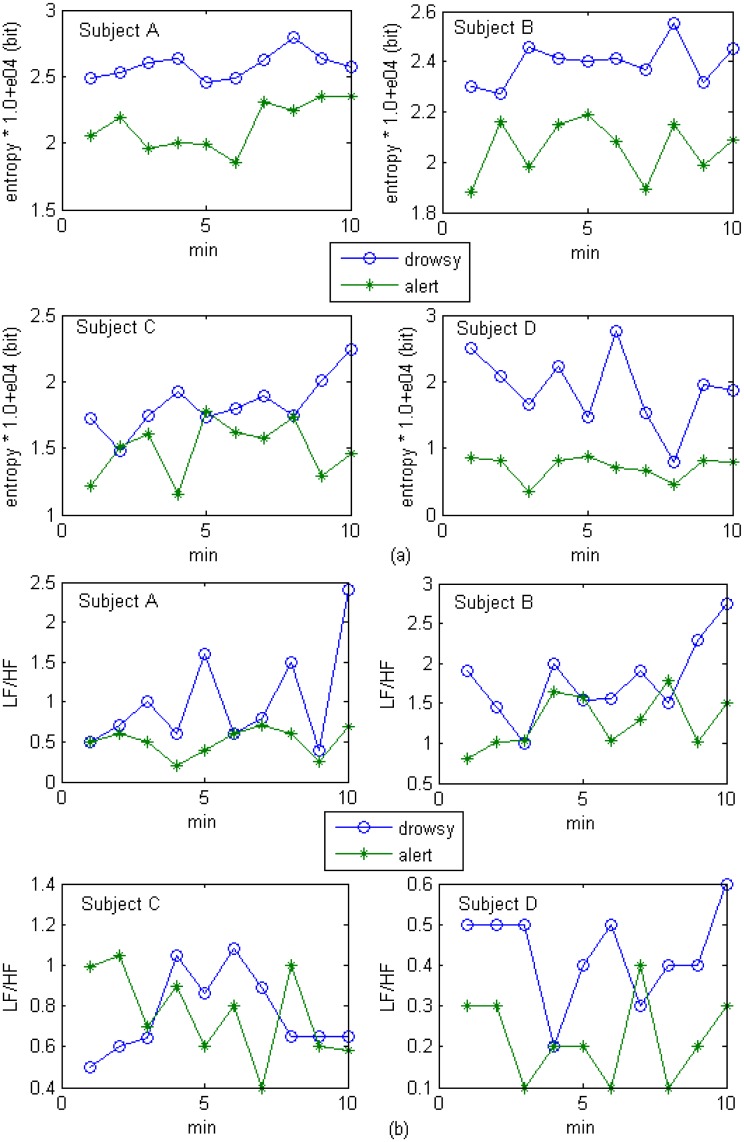
Entropy values of level A_8_ and LF/HF values in the drowsy driving and alert driving groups; (**a**) entropy values; (**b**) LF/HF values.

The changes for entropy and LF/HF values for 1-min HRV signals during 10-min alert state and drowsy state driving experiments are shown in Figure 11. The entropy and LF/HF values both increase when subjects are driving during a drowsy state. This result indicates the enhancement of SNS activities. However, individual differences are easy to recognize. For example, the female subject (subject D) had a lower entropy level during the alert state as compared to one of the male subjects (subject B), which indicates that the female subject was more relaxed during the alert state. However, her entropy values jumped to approximately 3 × 10^4^ bit (the maximum entropy among the four subjects) and then dropped to less than 1 × 10^4^ bit during the drowsy phase. This result indicates that the female subject was more nervous than the male subjects during the drowsy state. The LF/HF ratio is effective for classifying drowsy and alert states, but the overlap is obvious when comparing entropy levels. This point is demonstrated in Figure 10, where the ROC_area_ of entropy is higher than that of LF/HF ratio.

The statistic difference tests (independent *t*-test, *p* = 0.05) were also carried out for entropy and LF/HF values for 1-min HRV signals during 10-min alert state and drowsy state driving experimentsas shown in Figure 11. The test results are summarized in Table 7, where we can see that both wavelet-based feature (entropy) and FFT-based feature (LF/HF) have significant differences between alert and drowsy groups (except the FFT-based feature from subject C whose sig. (2-tailed) = 0.958 > 0.05). However, comparing the averaged sig. (2-tailed) values, we can find that wavelet-based feature [sig. (2-tailed) = 0.0005] is significantly better than FFT-based feature [sig. (2-tailed) = 0.3045].

The driver fatigue detection system should indentify drowsy driving conditions as early as possible. Since the wavelet-based feature (entropy at level A_8_) that is extracted from 1-min HRV signals is more powerful than the LF/HF that is based on 3-min HRV signals for both of male and female subjects, the 1-min entropy of level A_8_ was selected for training the SVM.

### 4.2. Classification Using SVM

Altogether, 80 LF/HF and entropy features from 40 drowsy and 40 alert samples are grouped into four datasets, each of which corresponds to a particular subject. Each dataset is composed of 20 entropy values, 20 LF/HF values, and 20 labels (the number of labels for alert and drowsy is 10 each). Each feature vector (entropy and LH/HF) and label in the dataset are denoted as *x_i_* and *L_i_* (*i* = 1, …, 20), respectively, and are used to train the SVM:

(9) $$\begin{array}{l} x_{i}=\{\text{entropy}\_\text{level}\_A8,LF/HF\}\\ L_{i}=\{\text{drowsy}(\text{true}),\text{alert}(\text{false})\} \end{array}$$

The LOO validation method is used to test the SVM classifier. The LOO method is a standardized approach for the validation of a classifier, where each feature vector serves as a test sample. The specific steps are as follows: (1) Omit a single feature vector from the dataset; (2) Train the classifier; (3) Test the omitted feature vector; (4) Repeat the steps that are listed above until each feature vector has been omitted and tested once. The LOO classification performance of SVM classifier is shown in Figure 8. Accuracy (Ac), sensitivity (Se), and specificity (Sp) are calculated as shown in Equation (10), where TP is true positive, TN is true negative, FP is false positive, and FN is false negative:

(10) $$\begin{array}{l} \text{Accuracy}=\frac{TP+TN}{TP+TN+FP+FN}\\ \text{Sensitivity}=\frac{TP}{TP+FN}\\ \text{Specificity}=\frac{TN}{TN+FP} \end{array}$$

**Figure 12.**
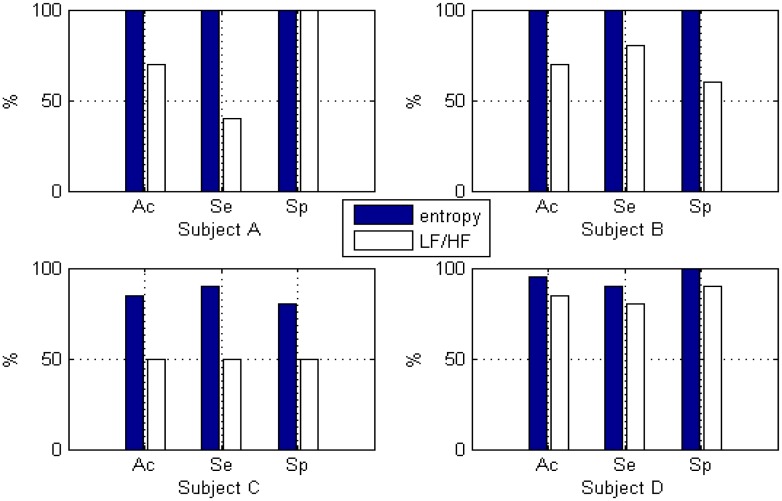
Performance of SVM classifier when using leave-one-out classification.

The best classification result using γ = 0.1 and *C* = 1 was obtained and shown in Figure 12, where we can see that the entropy measurement performs better than the LF/HF measurement for all of the subjects.

The best classification performances for entropy occur with subjects A and B with 100% accuracy, 100% sensitivity, and 100% specificity. This is what we would expect, because the ROC_area_ of entropy for both subjects A and B is at the maximum, *i.e.*, 1.00. The best classification performance for LF/HF occurs with subject D with an accuracy of 90%, a sensitivity of 85%, and a specificity of 95%. This is also what we would expect, because the ROC_area_ for LF/HF for subject D is 0.79, which is the highest value for the four subjects. Based on this classification results, we also found that ROC_area_ is a much better feature selection method compared to *t*-test. For example, for subject B in Table 7, *t*-test does not show any difference between wavelet-based feature and FFT-based feature because the sig. (2-tailed) value is the same zero, however ROC_area_ in Figure 10a is able to illustrate the difference (ROC_area_ for wavelet-based feature = 1.00, ROC_area_ for FFT-based feature = 0.73), which follows the higher classification accuracy (100%) for wavelet-based feature and lower accuracy (70%) for FFT-based feature.

## 5. Conclusions

The standard shortest time for LF/HF analysis on HRV is 2 min. In order to reduce the processing time and increase the real-time performance of driver drowsiness detection system, the feasibility of using wavelet-based features from shorter durations of PPG-derived heart rate variability data was tested. The FFT-based feature (LF/HF ratio) and the wavelet-based feature (entropy at level 8 of approximate coefficient) based on 1-min HRV segments were used for training the support vector machine classifier. The averaged performance for leave-one-out classification for the wavelet-based feature achieved 95% accuracy, 95% sensitivity, and 95% specificity. In contrast, the averaged performance for conventional LF/HF ratios is 68.8% accuracy, 62.5% sensitivity, and 75% specificity. This classification results indicate that a better real-time driver drowsiness detection system can be developed by using wavelet-based feature. In addition, the proposed system is inexpensive and easy-to-use. The main features included:

- A single PPG sensor node that was easy to attach to the steering wheel.
- Easy-to-use monitoring via smartphone.
- Tele-monitoring that was achievable via Internet.
- Built-in alertness boosting solution. This feature was based on Google Map and the Place API and displayed the location of the nearest coffee shop.

## Figures and Tables

**Table 1.** Specifications of fabricated PPG wireless sensor node.

**Components**	**Items**	**Specifications**
PPG sensor (Reflection type) [24]	LED	660 nm infrared
	Bandwidth	0.2 to 5.6 Hz
MCU [25]	ADC	10 bit
	Sampling rate	40 Hz
Bluetooth module [26]	Size	1.75 × 0.65
	Maximum transmission range	106 m
	Averaged power consumption	75 mW, at 3.3 V
Power	Battery	4.5 V

**Table 2.** HRV extraction from PPG raw data in smartphone.

**Procedure**		**Purpose**
**Step 1:** 1st-order differential operation	Remove artifacts caused by the driver's movements on the steering wheel	
**Step 2:** Peak-to-peak detection	Calculate P-P intervals	
**Step 3:** Re-sampling the P-P intervals at 7 Hz using cubic spline interpolation		

**Table 3.** Summary of the two different driving conditions.

**Driving conditions**	**Description Specifications**	**PERCLOS**
Alert	Driving in the morning at 9:00∼11:00	0% to 30%
Drowsy	Driving while attempting to remain alert, when feeling sleepy at 1:30∼2:30 am.	30% to 40%

**Table 4.** Comparison of the frequency ranges of the decomposition at each level and the conventional FFT-based HRV frequency domain.

**Wavelet Decomposition** **Level**	**Frequency Range (Hz)**	**FFT-based HRV frequency domain**
**A_8_**	0.005–0.01	**VLF** (0.003–0.04 Hz)
**D_8_**	0.01–0.03	

**D_7_**	0.03–0.05	**LF** and **HF** (0.04–0.4 Hz)
**D_6_**	0.05–0.11	
**D_5_**	0.11–0.22	
**D_4_**	0.22–0.44	

**D_3_**	0.44–0.88	–
**D_2_**	0.88–1.75	
**D_1_**	1.75–3.5	

**Table 5.** Summary of the subjects' data.

**Subjects' details**					**Data collected**	

**Subject**	**Age**	**Sex**	**Heart disease**	**Hypertensive**	**Alert (Min)**	**Drowsy (Min)**
A	28	Male	No	No	10	10
B	28	Male	No	No	10	10
C	26	Male	No	No	10	10
D	33	Female	No	No	10	10

**Table 6.** Example of the ROC areas for all 43 wavelet-based features based on 1-min HRV durations.

	**Level**	**Shannon Entropy**	**Variance**	**Kurtosis**	**Mean**	**Multiscale component (β)**		

	**A_8_**	***1.00***	0.54	***0.79***	***1.00***			
Subject A (28, male)	**D_8_**	0.38	0.36	***0.87***	0.42	D_8_, D_7_	β_256-128_	***0.71***
	**D_7_**	0.63	0.18	***0.77***	0.66	D_7_, D_6_	β_128-64_	0.69
	**D_6_**	0.36	0.09	0.69	0.51	D_6_, D_5_	β_64-32_	0.50
	**D_5_**	0.50	0.21	0.60	0.48	D_5_, D_4_	β_32-16_	0.49
	**D_4_**	0.50	0.48	0.61	0.54	D_4_, D_3_	β_16-8_	0.47
	**D_3_**	0.50	0.50	***0.73***	0.48	D_3_, D_2_	β_8-4_	0.46
	**D_2_**	0.50	0.50	***0.80***	0.50	D_2_, D_1_	β_4-2_	0.55
	**D_1_**	0.50	0.50	***0.76***	0.50	–	–	–

**Table 7.** Statistical significance tests results.

**Subject**	**Sig. (2-tailed)**	
	**Wavelet-Based Feature (Entropy)**	**FFT-Based Feature (LF/HF)**
A	0.000	0.026
B	0.000	0.000
C	0.002	0.958
D	0.000	0.000
